# Structural basis for the specific inhibition of glycoprotein Ibα shedding by an inhibitory antibody

**DOI:** 10.1038/srep24789

**Published:** 2016-04-22

**Authors:** Yue Tao, Xiaoqin Zhang, Xin Liang, Jianye Zang, Xi Mo, Renhao Li

**Affiliations:** 1 Key Laboratory of Pediatric Hematology & Oncology Ministry of Health, Pediatric Translational Medicine Institute, Shanghai Children’s Medical Center, Shanghai Jiao Tong University School of Medicine, Shanghai, China; 2 Aflac Cancer and Blood Disorders Center, Department of Pediatrics, Emory University School of Medicine, Atlanta, GA, USA; 3 Hefei National Laboratory for Physical Sciences at Microscale and School of Life Sciences; University of Science and Technology of China, Hefei, China; 4 Key Laboratory of Structural Biology; Chinese Academy of Sciences, Hefei, China

## Abstract

Ectodomain shedding of glycoprotein (GP) Ibα is thought to mediate the clearance of activated, aged or damaged platelets. A monoclonal antibody, 5G6, has been developed recently to specifically bind to the GPIbα shedding cleavage site and to inhibit its shedding. However, the molecular mechanism underlying antigen recognition and inhibitory specificity is not clear. To elucidate the structural basis for 5G6 binding to GPIbα, we determined the crystal structure of 5G6 Fab fragment in complex with its epitope peptide KL10 (GPIbα residues 461–470, KLRGVLQGHL), to 2.4-Å resolution. Key residues in both 5G6 and KL10 were mutated to validate their effects in antibody binding by using isothermal titration calorimetry. The 5G6 Fab-KL10 peptide complex structure confirmed the direct association of 5G6 with its target GPIbα residues and elucidated the molecular basis underlying its binding specificity and high affinity. The similar binding properties of 5G6 Fab fragment to GPIbα on human platelets as those to KL10 suggests that such an interaction may not be affected by the plasma membrane or nearby GPIbβ. This structural information may facilitate further antibody optimization and humanization.

In the case of elevated local blood shear stress, almost always due to vessel injury, the rapid adhesion and aggregation of circulating platelets to the vessel matrix is a critical step in hemostasis^1^,^2^. The GPIb-IX complex, the second most abundant receptor complex on the platelet surface, mediates a crucial step in platelet adhesion by interacting with its ligand von Willebrand factor (vWF)^3^. The GPIb-IX complex consists of three subunits, GPIbα, GPIbβ and GPIX, of which one molecule of GPIbα links with two molecules of asymmetric GPIbβ via disulfide bonds to form GPIb, while GPIX tightly associates with GPIb via non-covalent bonds in a 1:1 stoichiometry^4^,^5^.

Binding of GPIbα to vWF induces signal transduction into the platelets across the membrane, resulting in platelet activation and subsequent platelet aggregation and thrombus formation^6^,^7^,^8^. As a consequence of vWF binding, the surface density of GPIbα is rapidly decreased by releasing its ectodomain glycocalicin into the plasma, a process called ectodomain shedding. GPIbα ectodomain shedding is catalyzed by ADAM17 (a disintegrin and metalloproteinase 17), which cleaves GPIbα between Gly^464^ and Val^465 ^9,10. GPIbα is continuously shed in circulating platelets in a process that has been suggested to be a critical step in mediating the clearance of activated, aged or damaged platelets^11^,^12^. Additionally, the expression levels of GPIbα on the platelet surface correlate with the rolling speed and adhesion capacity of platelets. Therefore, the shedding of GPIbα after platelet activation may limit platelet reactivity, enhance the stability of formed thrombi, and regulate GPIbα association with αMβ2 on neutrophils and P-selectin on activated platelets^13^,^14^,^15^.

Previous studies have suggested that blocking GPIbα shedding with GM6001, a compound widely used to inhibit ADAM17 activity, would prevent the clearance of stored platelets^12^,^16^. However, because GM6001 is a broad-spectrum metalloproteinase inhibitor and ADAM17 is also a metalloproteinase with a broad substrate range, shedding of various other receptors on the platelet surface, such as GPV and GPVI, may also have been inhibited in these studies^17^,^18^. Therefore, to more accurately explore the effects of GPIbα shedding on platelet storage, six monoclonal antibodies that can specifically inhibit GPIbα shedding have been generated^19^. Among these antibodies, 5G6 and its monomeric Fab fragment (5G6 Fab) showed the highest binding affinity for purified GPIb-IX complex and inhibited GPIbα shedding with a similar potency as that of GM6001 without affecting the shedding of other receptors, platelet activation or aggregation^19^.

To elucidate the structural basis underlying antigen recognition and the high binding affinity of 5G6 for GPIbα, we solved the crystal structure of 5G6 Fab in complex with its epitope peptide KLRGVLQGHL (hGPIbα residues 461–470, short for KL10) at 2.4-Å resolution. Comparison of our structure with the structures of other Fab fragments revealed vital features of antigen recognition by 5G6 Fab, which were further confirmed by mutagenesis of 5G6Fab and KL10. In summary, we identified the features of the high binding affinity of 5G6 Fab for KL10 and shed light on the mechanisms of antibody-mediated shedding inhibition.

## Materials and Methods

### Production of 5G6 Fab fragment from hybridoma cells

5G6 hybridoma cells were cultured in Dulbecco’s Modified Eagle’s Medium (DMEM) supplemented with 10% fetal bovine serum (FBS), 2% L-glutamine, 0.5% penicillin/streptomycin, 1% hybridoma cloning factor, 0.5% hypoxanthine supplement and 1% non-essential amino acids. After the cell supernatant was collected, the monoclonal antibody (mAb) was purified using protein G beads (Invitrogen, Carlsbad, CA, USA). Then, 5G6 Fab fragment was prepared from 5G6 as previously described^19^. All Fab stock solutions were prepared in sterile phosphate-buffered saline (PBS) and stored at 4 °C until use.

### Crystallization and structural determination

Purified 5G6 Fab from hybridoma cells was mixed with the epitope peptide KL10 at a 1:2 molar ratio in crystallization solution (50 mM NaCl, 20 mM Tris·HCl, pH 7.4) and concentrated to approximately 10 mg/mL using a membrane with a 10-kDa molecular weight cut-off. After the initial screening, the complex was crystallized by the sitting drop method at 16 °C in 1.6M (NH_4_)_2_SO_4_, 0.01M NiCl_2_, and 0.1M Tris·HCl, pH 8.5. Single crystals were transferred to the same solution containing 20% (v/v) glycerol and were flash-frozen in liquid nitrogen. X-ray diffraction data were collected at 100 K at the Shanghai Synchrotron Radiation Facility (beamline BL17U1). The collected data were integrated and scaled using the program HKL-2000^20^. The complex structure was determined by molecular replacement with the structure of mAb 1479 (PDB code: 3U9U) as a search model^21^. Most of the residues in 5G6 Fab were located correctly, and the remaining portion was built manually using the program COOT^22^. The model was refined to 2.38 Å using the program sphenix.refine^23^ and COOT alternatively. The final model was validated using MolProbity^24^. Crystallographic statistics are listed in Table 1. All structural figures were prepared using PyMOL.

### Cloning, expression and purification of recombinant Fab fragment

Total mRNA was extracted from frozen 5G6 hybridoma cells, and reverse transcription PCR was performed to amplify the variable regions of the antibody. The cDNA fragments encoding the heavy and light chains of wild-type 5G6 Fab were cloned into a pcDNA3.1 vector with a C-terminal 6 × His tag (Invitrogen) and were then sequenced. Both vectors were cotransfected at a 1:1 ratio into suspended CHO cells in serum-free medium (Wuxi App Tec). Then, the transiently transfected cells were cultured at 37 °C with 6% CO_2_ and shaken at 125 rpm in disposable flasks. Seven days after transfection, the culture medium was collected and filtered, and the Fab proteins in the media were purified using a Ni-NTA Superflow column (Qiagen) and then further purified using a Hiload 16/60 Superdex 200 column (GE Healthcare) with Buffer A (200 mM NaCl, 20 mM Tris·HCl, pH 8.0). The eluted protein was collected and concentrated for isothermal titration calorimetry (ITC) and flow cytometry experiments.

### Isothermal titration calorimetry (ITC)

ITC was used to measure the binding affinities of various Fab fragments with KL10 peptide and wild-type 5G6 Fab fragment with various KL10 peptides. All Fab protein samples were purified in Buffer A at a final concentration of 0.02 mM, and all peptides were dissolved in Buffer A at 0.4 mM. The samples were centrifuged before the experiments to remove any protein precipitate. All measurements were performed at 25 °C using a MicroCal iTC200. Various KL10 peptides were titrated into the experimental cell that contained various Fab fragments in twenty 2 μL-injection increments. The titration data were analyzed using the ORIGIN data analysis software (MicroCal Software).

### Flow cytometry

Human platelets were seeded into a 96-well plate (Corning 3365, 1.5 × 10^6^ cells/well, 50 μL/well), incubated with various 5G6 Fab fragments at room temperature for 20 min, labeled with fluorescein isothiocyanate (FITC)-conjugated goat anti-mouse IgG, and washed with PBS buffer when necessary. The binding of Fab fragments was analyzed on a Becton-Dickinson FACS Canto II instrument (BD Biosciences, San Jose, CA, USA). The signal was quantitated by mean fluorescence intensity (MFI) for the entire cell population (10,000 cells).

## Results

### Crystal structure of 5G6 Fab in complex with the epitope peptide

**Figure 1:**
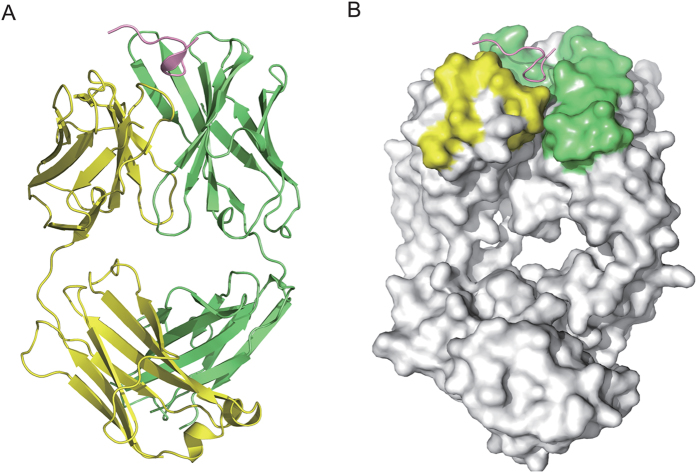
The crystal structure of the 5G6 Fab and epitope peptide KL10complex. (**A**) An overview of the complex structure. The 5G6 Fab heavy chain, light chain and the peptide are colored lime, yellow and pink, respectively. (**B**) The epitope peptide backbone is shown in the ribbon diagram, and 5G6 Fab is shown as a gray surface representation, except for the CDR loops that are in direct contact with the peptide. The CDR loops from the heavy chain are colored lime, and those from the light chain are yellow.

To elucidate the structural basis for the binding of 5G6 to the GPIbα shedding cleavage site, we determined the crystal structure of 5G6 Fab fragment in complex with its epitope peptide to 2.4-Å resolution (Table 1). The N-terminally acetylated epitope peptide contains 10 residues, KLRGVLQGHL, corresponding to residues 461–470 of human GPIbα (referred to as KL10). The crystal belongs to the space group C222_1_, with two Fab-peptide complexes in each asymmetric unit. The two complexes in the unit take on very similar structures and have a root-mean-square deviation (RMSD) value of 0.5 Å. In the complex (Fig. 1), 5G6 Fab adopts the canonical structure of IgG1 and belongs to the C_H_1/C_L_κ subtype, with 4 of the 6 CDR (complementarity-determining region) loops conforming to canonical classes, as defined by Chothia and colleagues^25^. The epitope peptide is not fully extended in the complex structure and covers the interface with CDR loops from both the heavy and light chains of 5G6. Notably, 5G6 Fab binds only to one side of the epitope peptide without obstructing both termini and it interacts with the entire peptide at an approximate right angle. Thus, it is very likely that the peptide conformation revealed in the complex structure may mimic the native conformation adopted by the same sequence as an accessible part of the mechanosensitive domain (MSD) in GPIbα^26^ and that binding of 5G6 to GPIb-IX may not be hindered by the juxtaposition of its binding epitope with the plasma membrane.

### Interface

**Figure 2:**
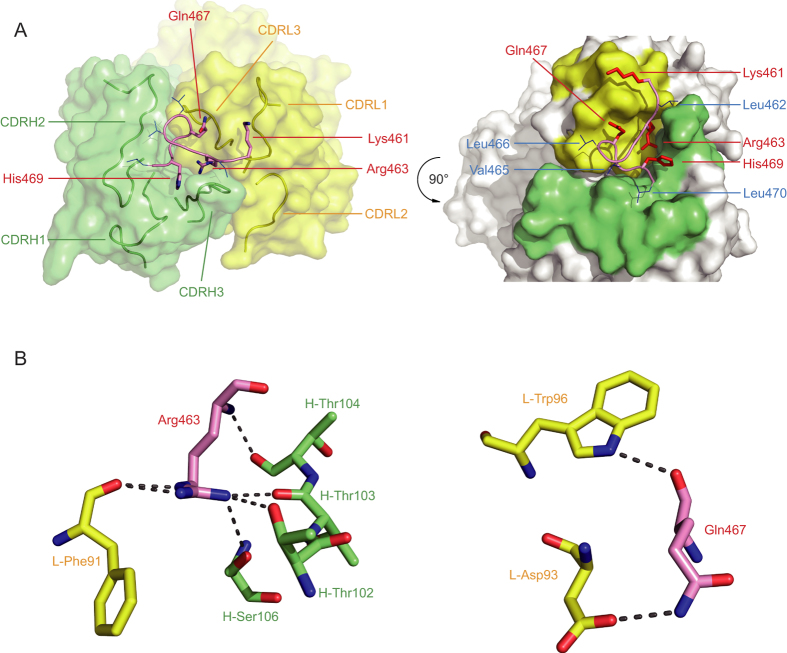
Detailed view of the epitope peptide KL10 bound to the 5G6 Fab fragment. (**A**) The interaction between 5G6 CDRs and KL10. Left panel: shown as a transparent molecular surface of 5G6 Fab with ribbon representations of the six CDRs. The polar residues in the epitope peptide, Lys^461^, Arg^463^, Gln^467^ and His^469^, are shown as sticks, and the remaining residues are shown as ribbons with side chains shown as blue lines. Right panel: side chains of polar residues in the peptide are shown as red thick sticks and the hydrophobic residues are shown as blue thin sticks. CDRH2 and CDRH3 are colored lime, whereas CDRL1 and CDRL3 are colored yellow. (**B**) Interaction of Arg^463^ and Gln^467^ in KL10 with 5G6. Protein and peptide are shown in stick representation with oxygens in red and nitrogens in blue. The dotted lines indicate hydrogen bonds. Coloring is the same as in Fig. 1.

Close inspection of the complex structure yielded the following observations about the binding interface between 5G6 Fab and the epitope peptide (Fig. 2A). First, the CDR loops of 5G6 form a groove at the binding interface. Over the groove residues, Gly^464^-Gln^467^ of the epitope peptide adopts a helix-like structure. As a result, the shedding cleavage site, the Gly^464^-Val^465^ peptide bond^10^,^27^, is not in direct contact with 5G6. Second, the Fab-peptide interface spans 647.7 Å^2^, of which 350.8 Å^2^ is with the heavy chain and 296.9 Å^2^ is with the light chain (as calculated using PISA and PDBsum, respectively)^28^,^29^. The extensive interface helps explain the high binding affinity of 5G6 for its epitope. Third, all polar residues in the epitope peptide, Lys^461^, Arg^463^, Gln^467^ and His^469^, are located on the same side of the peptide that is in direct contact with 5G6 Fab, and they constitute the majority of the binding interface (Fig. 2A). In particular, Arg^463^ and Gln^467^ supply atoms for 8 pairs of hydrogen bonds and hydrophobic contacts with 5G6 (Fig. 2B). It is notable that these two residues are specific for human GPIbα. Their murine counterparts are Pro and Leu, respectively, which may explain the inability of 5G6 to bind to murine GPIbα^19^. Fourth, it is also notable that the side chains of all hydrophobic residues in the epitope peptide, Leu^462^, Val^465^, Leu^466^ and Leu^470^, point away from the binding interface (Fig. 2A right panel), supporting the idea that several of these residues are part of the hydrophobic core of the juxtamembrane MSD of GPIbα^26^. Consistent with this notion, these hydrophobic residues are conserved in the murine sequence (Supplementary Fig. S1). Overall, the Fab-peptide complex structure confirmed the direct association of 5G6 with its target GPIbα residues and elucidated the molecular basis underlying the binding specificity and high affinity.

### Structural basis underlying the high binding affinity

**Figure 3:**
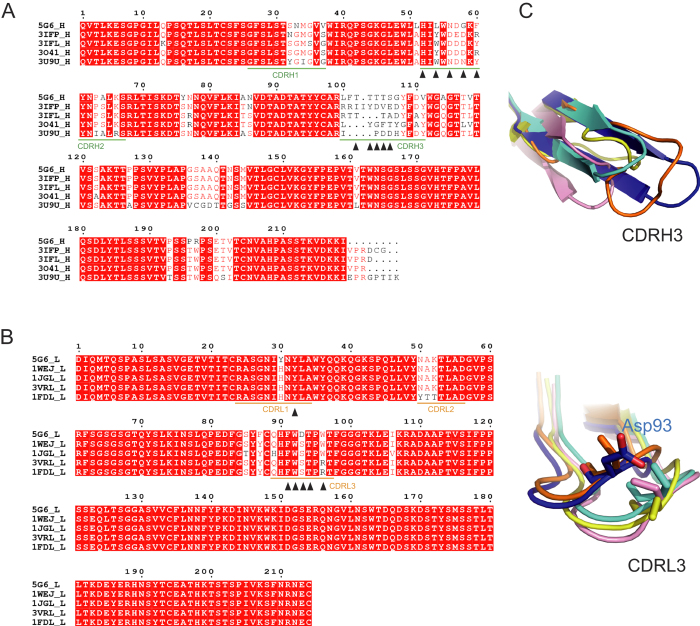
Sequence alignment and structural comparison of 5G6 Fab fragment. Sequence alignment of the 5G6 heavy chain (**A**) and light chain (**B**). The residues that are involved in the epitope peptide interaction are indicated by a black triangle under each residue. (**C**) Structural comparison of CDRH3 (upper panel) and CDRL3 (lower panel).

To fully elucidate the molecular basis underlying the binding specificity and high affinity of 5G6 for its epitope, sequence alignment of the heavy and light chains of 5G6 with several known antibodies was performed (Fig. 3A,B, respectively). Among the residues that participate in the interaction with KL10, CDRH3 and Asp^93^ of CDRL3 are not found in other antibodies but are specific to 5G6, whereas residues in CDRH2 and CDRL1 are either conserved in other antibodies or provide a hydrophobic environment. Structural comparison indicates that CDRH3 adopts a canonical β-sheet conformation with a relatively rigid loop region (Fig. 3C). In particular, the four threonine residues (Thr^102–105^) in this loop region provide atoms for 4 pairs of hydrogen bonds and hydrophobic contacts with KL10. Therefore, CDRH3 and Asp^93^ of CDRL3 may play decisive roles in the binding specificity and high affinity of 5G6 for GPIbα.

**Figure 4:**
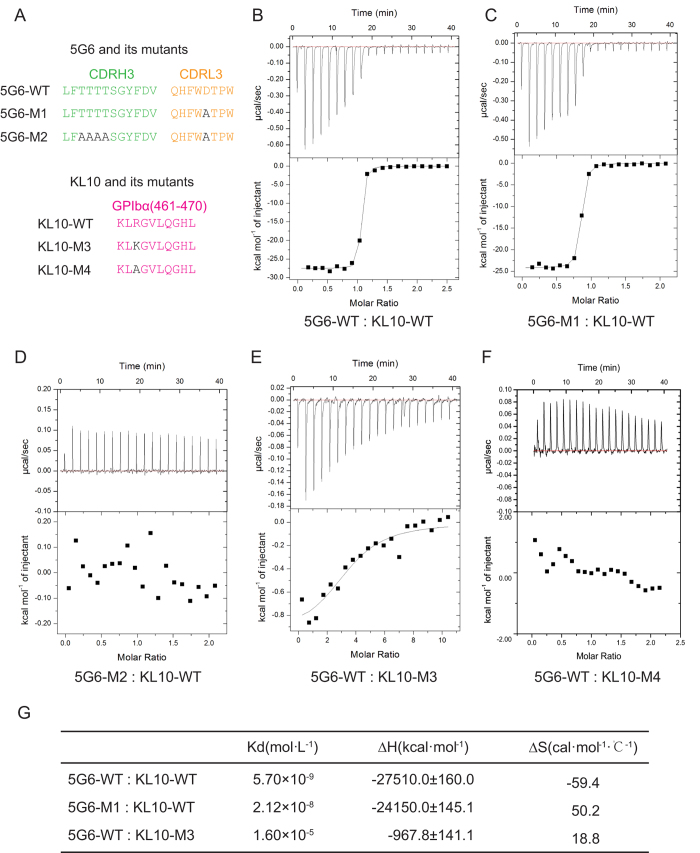
Binding affinity measurements of KL10 peptide/mutants and 5G6 Fab/mutants by ITC. ITC was performed by titrating KL10 peptide and its mutants into the experimental cell containing 5G6 Fab or its mutants. The top panel shows the heat change upon ligand titration. The bottom panel shows the integrated data and ITC isotherm (solid line) fitted to a single-site binding model. (**A**) Schematic drawing of the mutants used in ITC. ITC curves and fitted affinity of 5G6-WT: KL10-WT (**B**) 5G6-M1: KL10-WT (**C**) 5G6-M2: KL10-WT (**D**) 5G6-WT: KL10-M3 (**E**) and 5G6-WT: KL10-M4 (**F**). (**G**) Thermodynamic parameters from ITC studies.

To further validate that Thr^102–105^ and Asp^93^ were the key residues mediating 5G6 binding specificity and affinity, two mutant Fab fragments were generated, and their binding affinities for KL10 were determined using ITC. In the mutant 5G6 Fab 5G6-M1, Asp^93^ of CDRL3 was replaced with Ala, whereas in the mutant 5G6-M2, both the Thr^102–105^ of CDRH3 and the Asp^93^ of CDRL3 were replaced with Ala (Fig. 4A). To ensure the same origin of the Fab proteins, both the wild-type 5G6 Fab fragment and its mutants were expressed in CHO cells and purified by affinity chromatography. Consistent with results from our previous report^19^, the binding affinity of the wild-type 5G6 Fab fragment for KL10, measured by ITC, was 5.7 nM (Fig. 4B), approximately 4-fold higher than that of 5G6-M1 (21.2 nM, Fig. 4C). In contrast, 5G6-M2, with five point mutations in the interface, completely lost its capacity to interact with KL10 (Fig. 4D). These results demonstrated that Asp^93^ of CDRL3and Thr^102–105^ of CDRH3 play decisive roles in the binding specificity and high affinity of 5G6 for its epitope.

To verify the role of CDRH3, antibody 15C6, which contains a similar CDRH3 sequence, was studied. We previously reported that the affinity of 15C6 for GPIb-IX (~28 nM) was slightly weaker than that of 5G6 (~3 nM), but much higher than those of other antibodies^19^. Consistently, in the present study, the dissociation constant (K_d_) of 15C6 with KL10 measured by ITC assay was 35 nM. 5G6 and 15C6 differ by only seven amino acids, of which only three are in the CDR loops (Supplementary Fig. S2A). Sequence comparison revealed that Thr^102^ of 5G6 CDRH3 and Tyr^30^ of 5G6 CDRL1 are replaced by Ser and His, respectively, in 15C6. It was interesting to note that Tyr^30^ is specific to 5G6, but that His^30^ is conserved in 15C6 and the other four antibodies (Fig. 2B and Supplementary Fig. S2B). To test whether these two sites are responsible for the difference in 5G6 and 15C6 binding affinity, a T102S and Y30H double mutant (5G6-M5) was generated, and the mutant protein was expressed and purified (Supplementary Fig. S2A). 5G6-M5 exhibits a similar binding capacity as that of 5G6, suggesting that these two residues are not primarily responsible for the weaker affinity of 15C6 (Supplementary Fig. S2B). It is possible that the other three amino acids slightly alter the conformation of 15C6 and result in the fine distinction.

### Conformation of KL10

As shown in Fig. 2B, Arg^463^ of KL10 supplies atoms for 6 pairs of hydrogen bonds and hydrophobic contacts with 5G6. In particular, the Nη atom of Arg^463^ in the interface is extremely important. To confirm the role of Arg^463^ in epitope recognition, two mutant KL10 peptides were synthesized. In the mutant KL10 peptide KL10-M3 and KL10-M4, Arg^463^ was replaced by Lys and Ala, respectively (Fig. 4A). The binding affinities of these mutant peptides for the wild-type 5G6 Fab fragment were determined using the ITC technique. When Arg^463^ was replaced by Ala, the residue lacked the side chains involved in the interaction with 5G6, and thus, as expected, KL10-M4 had no interaction with 5G6 (Fig. 4F). In contrast, when Arg^463^ was replaced by Lys, the guanidine group in the side chain of Arg^463^ changed into an amino group that may supply similar pairs of hydrogen bonds as Arg^463^. However, to our surprise, the dissociation constant of KL10-M3 with 5G6 was approximately 1000-fold lower (16 μM, Fig. 4E) than the wild-type KL10 peptide (5.7 nM). These ITC results indicate that Arg^463^ is indispensable for the binding: it provides key elements for the interaction with 5G6 and may also be important for peptide conformation.

### 5G6 mutants have lower affinity to human platelets

**Figure 5:**
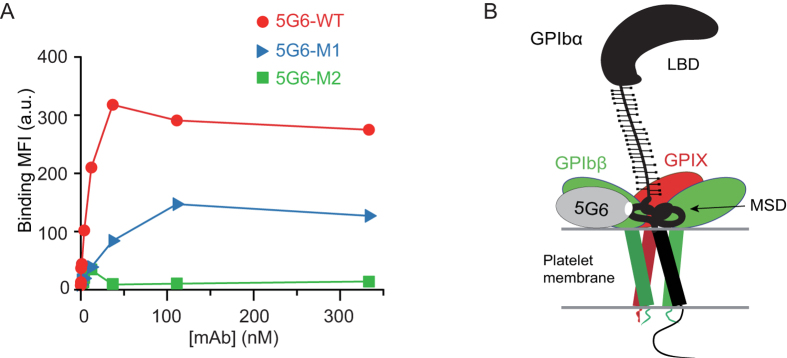
Binding of 5G6 Fab fragment and its mutants to human platelets and a cartoon model. (**A**) Binding plots of 5G6 Fab and its mutant proteins to human platelets. Human platelets were incubated with each mAb at the indicated concentrations for 20 min and were then washed once. Binding of mAb was detected by flow cytometry using FITC-conjugated goat anti-mouse IgG and was quantified according to the mean fluorescence intensity (MFI) of 10,000 cells. (**B**) A cartoon illustrating a model of 5G6 in complex with full-length GPIb-IX. The illustration is based on the relative orientations of 5G6 Fab and the epitope peptide in the complex structure.

Our previous study has revealed that both 5G6 and 5G6 Fab fragments exhibit strong binding to human platelets, as detected by flow cytometry^19^. To address whether the two mutant 5G6 Fab fragments 5G6-M1 and 5G6-M2 also have weaker affinities for human platelets as observed in ITC measurements, similar flow cytometry assays were performed described previously^19^. Compared to wild-type 5G6 Fab fragment, 5G6-M1 showed relatively lower affinity for human platelets, whereas 5G6-M2 had no binding (Fig. 5A). These results are consistent with ITC observations, because 5G6-M2 lost its capacity to interact with KL10 peptide (Fig. 4D).

## Discussion

GPIbα, a major receptor in platelets, is continuously cleaved from the platelet surface and releases its extracellular domain, glycocalicin, into the plasma, which has a glycocalicin concentration of 1–3 μg/mL^30^. Such cleavage, also known as GPIbα ectodomain shedding, has been reported since 1981 and is mediated by ADAM17^10^,^11^. In addition to constitutive shedding, GPIbα ectodomain shedding can be up-regulated under certain circumstances, such as platelet activation^14^. The biological significance of GPIbα shedding remains to be defined, but it has been suggested to serve as a biomarker for atherosclerosis, since an increased glycocalicin level has been reported in patients with atherosclerosis^31^. More importantly, GPIbα shedding has been suggested to be related to platelet production, storage and clearance^32^,^33^,^34^. Various studies have consistently reported that glycocalicin accumulates during platelet storage. Thus, GPIbα shedding is thought to be a specific characteristic of the platelet storage lesion, one of the major reasons for the short shelf-life of platelets^12^,^27^,^32^,^33^,^35^. Treatment with the broad-spectrum metalloprotease inhibitor GM6001 or inhibition of ADAM17 activity with p38 mitogen-activated protein kinase (MAPK) inhibitors improves murine platelet recovery during storage^12^,^16^. However, because ADAM17 has a broad range of substrates, these studies do not specifically target GPIbα shedding. Therefore, to explore the functions of GPIbα shedding, a monoclonal antibody, 5G6, that specifically inhibits GPIbα shedding was screened in our previous study^19^.

The extracellular domain of GPIbα consists of three regions: an N-terminal ligand-binding domain (LBD) for the interaction with vWF and other ligands, a sialomucin domain that extends for tens of nanometers, and a quasi-stable MSD (Fig. 5B)^26^,^36^,^37^. The MSD, which harbors the GPIbα shedding cleavage site, is structured but relatively unstable. It is postulated to undergo unfolding upon ligand binding and mechanical pulling, leading to transmembrane signaling^26^. Because the proper expression of GPIbα on the platelet membrane requires its correct association with GPIbβ and GPIX, it has been hypothesized that the folded MSD in GPIbα is surrounded and constrained by its neighboring GPIbβ and GPIX subunits, leaving a potential entry site for ADAM17^19^,^26^. This entry site for ADAM17, which is demonstrated can be blocked by 5G6 in the previous study, is supposed to be available when MSD is unstructured^19^,^26^. Because both the folded MSD in the full length GPIb-IX complex and KL10 can be recognized by 5G6 and bound at similar affinity, the structure of KL10 in 5G6 Fab-KL10 complex may partly provide the structural information of MSD in its native condition. In the complex structure, KL10 adopts a partially extended conformation with four polar residues (Lys^461^, Arg^463^, Gln^467^ and His^469^) constituting the majority of the binding interface and four hydrophobic residues (Leu^462^, Val^465^, Leu^466^ and Leu^470^) pointing away from the interface. Therefore, Leu^462^, Val^465^, Leu^466^ and Leu^470^ in KL10 are suggested to be part of the native MSD structure.

5G6 Fab shares high sequence homology with four other antibodies and adopts the canonical structure of IgG1. Sequence alignments show that CDRH1 and CDRH2 of 5G6 are conserved among different antibodies, indicating their limiting roles in binding specificity. In contrast, CDRH3 is specific to 5G6 and accounts for the majority of the hydrogen bonds between 5G6 and KL10. In the complex structure, Thr^102–105^ in CDRH3, along with Asp^93^ in CDRL3 were the key residues for 5G6 in mediating binding specificity and affinity, while Arg^463^ was the corresponding key residue for peptide KL10 (Fig. 2B). To further validate this hypothesis, two mutants for 5G6 Fab and two mutants for KL10 were generated and applied to ITC experiments (Fig. 4). In 5G6-M1, where Asp^93^ in CDRL3 was mutated to alanine, only the corresponding hydrogen bond between CDRL-Asp^93^ and KL10-Gln^467^ was lost, with the main hydrogen bonds and hydrophobic interactions remained. Therefore, only a subtle change in ∆H and ∆S was caused, and as a result a 3-4 fold of decrease in its binding affinity to KL10. In contrast, when the four threonine residues 102–105 were also mutated to alanine in 5G6-M2, the hydrogen bonds between CDRH-Thr^102^ and KL10-Arg^463^, KL10-Leu^466^, the hydrogen bond between CDRH-Thr^103^ and KL10-Arg^463^, as well as the hydrogen bond between CDRH-Thr^104^ and KL10-Arg^463^ were all absent, abolishing the binding of the Fab protein to KL10. On the other hand, guanidine group of Arg^463^ in KL10, responsible for two hydrogen bonds with CDRL-Phe^91^ and one hydrogen bond each with CDRH-Thr^102^, Thr^103^ and Ser^106^, plays a pivotal role in epitope recognition. When Arg^463^ was replaced by a lysine in KL10-M3, the corresponding amine group in lysine failed to sustain the two hydrogen bonds with CDRL-Phe^91^ and the other hydrogen bonds with CDRH-Thr^102^, Thr^103^ and Ser^106^ were also supposed to be compromised, leading to a significant increase of ∆H and a nearly 3000-fold decrease in its binding affinity with 5G6. Additionally, the orientation of KL10-M3 with 5G6 might be deviated compared to KL10-WT, and the hydrophobic interactions between Arg^463^ and CDRL-Trp^92^ is likely to be attenuated as a consequence, resulting in significant increase of ∆S. When Arg^463^ was replaced by an alanine in KL10-M4, all the five hydrogen bonds mentioned above were supposed to be absent, which led to a complete loss of its capacity to interact with 5G6 as expected.

As indicated above, ITC data for the mutant Fab proteins demonstrated that CDRH3 is the key fragment in the binding affinity and specificity of 5G6 for its epitope (Fig. 4C,D). Moreover, the importance of Thr^102–105^ and Asp^93^ in CDRH3 in binding to the full length GPIbα expressed on the cell surface was also confirmed in flow cytometry studies (Fig. 5A). Therefore, our structural and biochemical data indicate the above crucial residues in the antigen recognition and high binding affinity of 5G6 for GPIbα and may provide the molecular basis for further antibody optimization and antibody humanization. Furthermore, because the specific inhibition of GPIbα shedding through treatment with 5G6 Fab markedly improves post-transfusion recovery *in vivo* (data unpublished), the data presented herein may facilitate the development of a new generation of GPIbα shedding inhibitors that may be utilized in platelet storage.

Overall, in the present study, we determined the 5G6 Fab-KL10 complex structure, which confirmed the direct association of 5G6 with GPIbα residues flanking the shedding cleavage site, and elucidated the molecular basis underlying the binding specificity and high affinity.

## Additional Information

**How to cite this article**: Tao, Y. *et al.* Structural basis for the specific inhibition of glycoprotein Ibα shedding by an inhibitory antibody. *Sci. Rep.* **6**, 24789; doi: 10.1038/srep24789 (2016).

## Supplementary Material

Supplementary Information

## Figures and Tables

**Table 1:** Crystallographic data collection and refinement statistics for the complex of 5G6 Fab with epitope peptide KL10 (4YR6).

Space group	C222_1_
Unit cell parameters	
a, b, c (Å)	101.87, 147.02, 194.87
α, β, γ (°)	90, 90, 90
Resolution (Å)^a^	50-2.38 (2.51-2.38)
NO. of unique reflections	57,791 (8,023)
Wilson plot B factors (Å^2^)	39.0
R_merg_(%)^b^	8.4 (45.2)
Mean I/σI	15.3 (3.8)
Completeness (%)	98.3 (94.7)
Redundancy	7.1 (6.2)
Refinement	
Resolution (Å)	2.38
R_work_^c^/R_free_^d^	21.24/25.38
R.m.s. deviations	
Bond lengths (Å)	0.008
Bond angles (°)	1.225
B-factors (Å^2^)	33.8
Protein	866
Water	290
Other ligand	22
Ramachandran plot	
Most favored regions (%)	96.3
Additionally allowed regions (%)	3.6
Outliers (%)	0

^a^The values in parentheses refer to statistics in the highest shell.

^b^*R*_*merge*_ = ∑_*hkl*_∑_*i*_|*I*_*i*_(*hkl*) − <*I*(*hkl*)> |/∑_*hkl*_∑_*i*_| *I*_*i*_(*hkl*)| where *I*_*i*_(*hkl*) is the intensity of the ithmeasurement, and <*I*(*hkl*)> is the mean intensity for that reflection.

^c^R_factor_ = ∑∑_hkl_||Fo|-|Fc||/∑∑_hkl_|Fo|, where |Fo| and |Fc| are the observed and calculated structure factor amplitudes, respectively.

^d^R_free_ was calculated with 5.0% of the reflections in the test set.
