# Supplementary material for: Structural basis for the specific inhibition of glycoprotein Ibα shedding by an inhibitory antibody
Source: Sci Rep. 2016 Apr 22;6:24789. doi: 10.1038/srep24789 (PMC4840364; doi:10.1038/srep24789)
Supplement: Supplementary Information [file srep24789-s1.pdf]

Structural basis for the specific inhibition of glycoprotein Ibα shedding by an inhibitory antibody

Yue Tao[1,#], Xiaoqin Zhang[1,#], Xin Liang[2], Jianye Zang[3,4], Xi Mo[1,*], Renhao Li[2,*]

[1]Key Laboratory of Pediatric Hematology & Oncology Ministry of Health, Pediatric Translational Medicine Institute, Shanghai Children's Medical Center, Shanghai Jiao Tong University School of Medicine, Shanghai, China

[2]Aflac Cancer and Blood Disorders Center, Department of Pediatrics, Emory University School of Medicine, Atlanta, GA, USA

[3]Hefei National Laboratory for Physical Sciences at Microscale and School of Life Sciences; University of Science and Technology of China; Hefei, China

[4]Key Laboratory of Structural Biology; Chinese Academy of Sciences; Hefei, China

Correspondence:

Renhao Li, Department of Pediatrics, Emory University School of Medicine, 2015 Uppergate Drive NE, Room 440, Atlanta, GA 30322, USA. Tel.: +1 404 727 8217; fax: +1 404 727 4859. E-mail: renhao.li@emory.edu.

Xi Mo, Pediatric Translational Medicine Institute, Shanghai Children's Medical Center, 1678 Dongfang Rd., Room 2146, Shanghai 200127, China. Tel.: +86 21 38626161*85290; fax: +86 21 58756923. E-mail: xi.mo@shsmu.edu.cn.

Addendum

[#]These authors contributed equally to this work.

Competing interests

The authors declare no competing financial interests.

Figure S1

```
H.sapiens(461-470)      KLRGVLQGHL
M.musculus(569-578)     NLPEVALVSS
                         ▲  ▲▲   ▲
```

Supplementary Figure S1. Sequence alignment of KL10 peptide. Residues 461-470 in human GPIbα and 569-578 in murine GPIbα are aligned. The hydrophobic residues are indicated by black triangles under the residues.

Figure S2

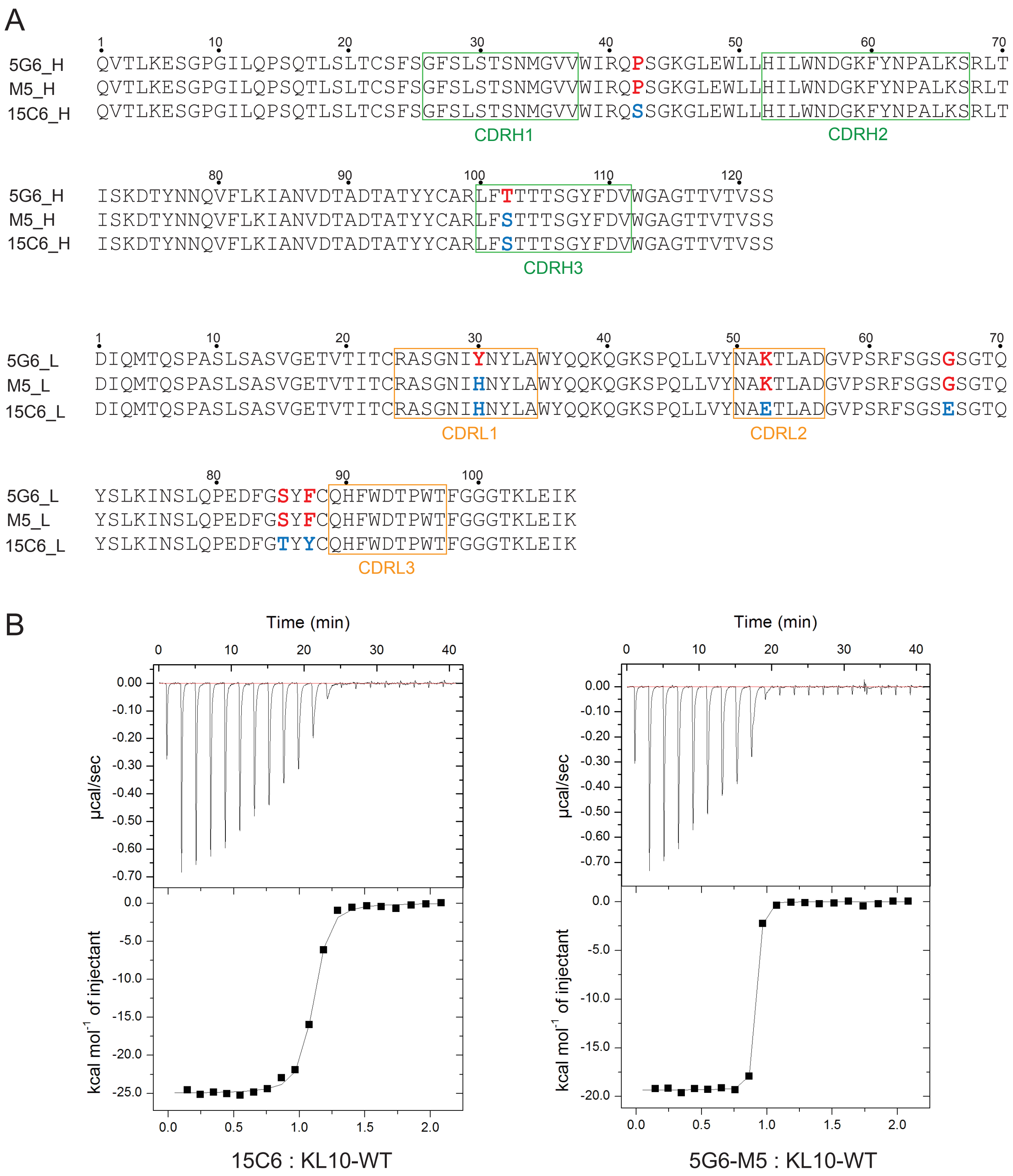

C

	Kd(mol·L^{-1})	ΔH(kcal·mol^{-1})	ΔS(cal·mol^{-1}·℃$^{-1}$)
15C6 : KL10-WT	3.50×10^{-8}	-25000.0±157.5	-54.2
5G6-M5 : KL10-WT	2.50×10^{-9}	-28430.0±196.8	-62.0

Supplementary Figure S2. Binding affinity measurement of KL10 peptide and 15C6 Fab/mutants by ITC. (A) Sequence alignment of 5G6, 15C6 and 5G6-M5. Different residues are colored red or blue. CDR loops are indicated with rectangles. (B) ITC was performed by titrating KL10 peptide into the experimental cell containing 15C6 Fab or 5G6-M5. The top panel shows the heat change upon ligand titration; the bottom panel shows the integrated data and ITC isotherm (solid line), fitted to a single-site binding model. (C) Thermodynamic parameters from ITC studies.

Figure S3

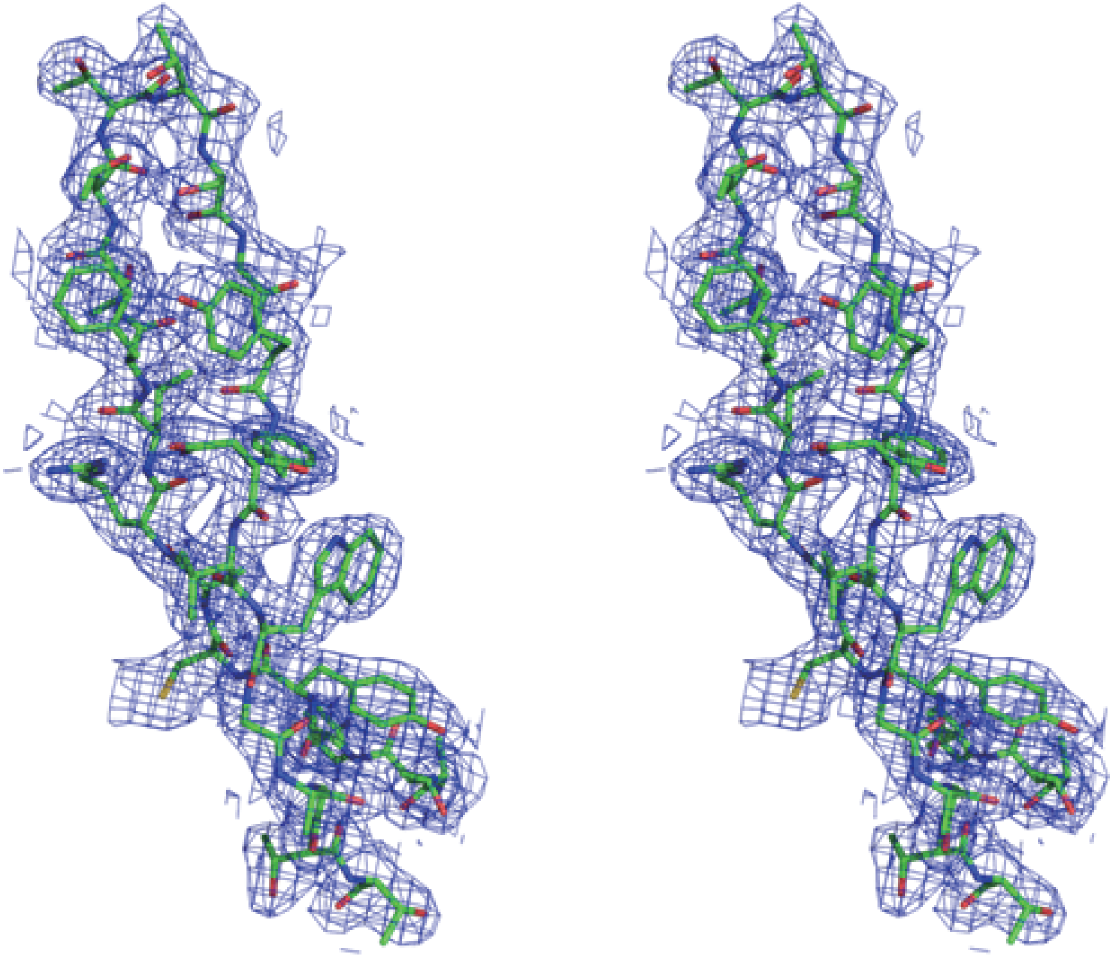

Supplementary Figure S3. A portion of the electron density map. The density map of Chain A (93-117) amino acids.
